# Molecular Detection of *Candidatus Anaplasma camelii* in Naturally Infected Dromedary Camels (*Camelus dromedarius*) in Abu Dhabi Emirate, United Arab Emirates, 2019–2023

**DOI:** 10.3390/vetsci11030123

**Published:** 2024-03-07

**Authors:** Hassan Zackaria Ali Ishag, Shameem Habeeba, El Tigani Ahmed El Tigani-Asil, Mohd Farouk Yuosf, Zulaikha Mohamed Abdel Hameed Al Hammadi, Abraham Nii Okai Commey, Hashel Talal Aboud Amer Bin Hraiz, Asma Abdi Mohamed Shah, Abdelmalik Ibrahim Khalafalla

**Affiliations:** Biosecurity Affairs Division, Development and Innovation Sector, Abu Dhabi Agriculture and Food Safety Authority, Abu Dhabi P.O. Box 52150, United Arab Emirates

**Keywords:** detection, *Candidatus Anaplasma camelii*, *Camelus dromedarius*, *groEL*, phylogenetic, United Arab Emirates

## Abstract

**Simple Summary:**

Camel anaplasmosis is a recent emerging disease with potential zoonotic concerns. There is poor understanding of the epidemiology of anaplasmosis in camels and, in particular of *Candidatus Anaplasma camelii*, which is detected in several countries including Saudi Arabia, Iran, Kenya, and Morocco. Most studies of anaplasmosis in camels relied on microscopy and serology for diagnosis, and few used molecular approaches. The present work characterizes Anaplasmataceae strains circulating in the *Camelus dromedarius* reservoir in the United Arab Emirates (UAE) using PCR, sequencing, and phylogenetic analysis for the first time to provide information about the largely neglected disease they cause. Between 2019 to 2023, thirty-five whole-blood samples (35/287 = 12.2%) tested positive for *Anaplasmataceae* spp. by PCR assay targeting the *groEL* gene. Of these, only nine positive samples (9/35 = 25.7%) were sequenced using *groEL* gene primers. A GenBank BLAST analysis revealed that all strains were 100% identical to the *Candidatus Anaplasma camelii* reference sequence available in the GenBank nucleotide database.

**Abstract:**

The recent emergence of anaplasmosis in camels has raised global interest in the pathogenicity and zoonotic potential of the pathogen causing it and the role of camels as reservoir hosts. In the United Arab Emirates (UAE), molecular studies and genetic characterization of camel-associated Anaplasma species are limited. This study aimed to characterize molecularly Anaplasmataceae strains circulating in dromedary camels in the UAE. Two hundred eighty-seven whole-blood samples collected from dromedary camels across regions of the Abu Dhabi Emirate were received between 2019 and 2023 at the Abu Dhabi Agriculture and Food Safety Authority (ADAFSA) veterinary laboratories for routine diagnosis of anaplasmosis. The animals were sampled based on field clinical observation by veterinarians and their tentative suspicion of blood parasite infection on the basis of similar clinical symptoms as those caused by blood parasites in ruminants. The samples were screened for Anaplasmataceae by PCR assay targeting the *groEL* gene. Anaplasmataceae strains were further characterized by sequencing and phylogenetic analysis of the *groEL* gene. Thirty-five samples (35/287 = 12.2%) tested positive for *Anaplasmataceae* spp. by PCR assay. Nine positive samples (9/35 = 25.7%) were sequenced using *groEL* gene primers. GenBank BLAST analysis revealed that all strains were 100% identical to the *Candidatus A. camelii* reference sequence available in the GenBank nucleotide database. Phylogenetic analysis further indicated that the sequences were close to each other and were located in one cluster with *Candidatus A. camelii* sequences detected in Saudi Arabia, Morocco, and the UAE. Pairwise alignment showed that the UAE sequences detected in this study were completely identical and shared 100% identity with *Candidatus A. camelii* from Morocco and Saudi Arabia and 99.5% identity with *Candidatus A. camelii* from the UAE. This study demonstrates the presence of *Candidatus A. camelii* in UAE dromedary camels. Further critical investigation of the clinical and economical significance of this pathogen in camels needs to be carried out.

## 1. Introduction

The dromedary, or one-humped camel (*Camelus dromedarius*), is an adaptable animal that can survive in stringent ecological conditions across a range of dry, semi-arid, and tropical regions of Asia, Africa, and Australia and still produce milk and meat for human consumption. In the Middle East and North Africa’s harsh desert regions, dromedary camels used to be the only method of transportation, supporting survival and long-term life. Millions of people rely on camels for their daily needs and subsistence in the pastoral regions of central Asia and North and East Africa [1]. Additionally, dromedaries have particular significance in the Arab-Islamic culture. They are utilized in tourism, beauty pageants, and racing and represent a national wealth and source of income, especially in the Gulf countries. There has been a rise in the interest in improving camel health and productivity due to the rising global warming and the camels’ ability to survive under harsh climatic conditions [2].

Camels were previously thought to be resistant to most diseases that generally affect livestock because they thrive in harsh and arid conditions. However, further investigations revealed that camels are vulnerable to various pathogens.

Hemoprotozoans are blood-borne, unicellular eukaryotes that can infect any class of terrestrial vertebrate groups [3]. The major tick-transmitted diseases that attract the most attention are ehrlichiosis, theileriosis, anaplasmosis, and babesiosis. Anaplasmosis is a disease caused by *Anaplasma* spp., zoonotic obligate intracellular parasites transmitted by ticks and other arthropods. It is caused by *Anaplasma* spp., which belongs to the Anaplasmataceae family [4] that includes six genera, i.e., Ehrlichia, Anaplasma, Aegyptianella, Wolbachia, Neorickettsia, and Candidatus Neoehrlichia [5]. The disease occurs in tropical and subtropical regions of America, Europe, Africa, Asia, and Australia [6,7,8]. Anaplasmosis, also called Gall sickness or tick-borne fever, affects a wide range of hosts including cattle, sheep, goats, buffalo, and some wild ruminants [9,10]. Various Anaplasma species within the genus Anaplasma (denoted as A.), such as *A. marginale*, *A. centrale*, *A. ovis*, *A. bovis*, *A. platys*, and *A. phagocytophilum*, have been reported to infect livestock, and some are zoonotic, as they can infect humans [11,12,13]. Anaplasmosis has a complex epidemiology due to its wide host range, the existence of different species of Anaplasma causing the disease, and vector transmission. In addition to the intraerythrocytic forms of Anaplasma, *A. bovis* causes intramonocytic anaplasmosis, and *A. platys* causes canine cyclic thrombocytopenia in dogs—infecting platelets—and equine granulocytic anaplasmosis in horses [14]. Anaplasmosis adversely affects livestock health and performance, causing an average cost of USD 793 per head of cattle, 54% of which can be attributed to death, followed by 15% attributable to disease treatment, 14% to weight loss, 8% to chronic disease, and 9% to abortion. So far, five species of Anaplasma have been detected in dromedary camels including *A. phagocytophilum* [15,16], *A. platys* [17,18], *A. ovis* [19], *A. marginale* [19,20], and *Candidatus A. camelii* (genetically close to *A. platys*), recently detected in dromedary camels in Saudi Arabia [21,22], Iran [23], Morocco [14], and Kenya [24,25]. However, their veterinary and economic importance in camel is still not well clarified, and more applied research is needed, especially in the fields of pathogenicity and pathogenesis, to uncover the clinical role of such parasites in camel health and production.

In camels, anaplasmosis usually appears as a subclinical infection or co-infection without any distinctive clinical signs. However, some researchers reported a clinical form of Anaplasmosis in one-humped camels with various signs, including fever, anemia, emaciation, slight ataxia, anorexia, jaundice, abortion, or enlargement of the lymph nodes [14,26,27,28]. The clinical diagnosis of anaplasmosis in camels is challenging due to its nonspecific clinical signs [29]. But it can primarily be diagnosed by microscopy, which is a lowly sensitive technique, and diagnosis heavily relies on the appropriate timing of sampling in the course of the disease, parasitemia, and the examiner’s skills [30]. Serology [31,32,33] or molecular methods targeting different genes, including 16S rRNA, 23S rRNA, and *groEL*, are more sensitive diagnostic tests for anaplasmosis in animals, as parasitemia can drop below the detectable limits of light microscopy [14,19,21,23,34,35,36].

In the Middle East, anaplasmosis in camel, caused specifically by *Candidatus A. camelii*, has been detected in several countries such as Saudi Arabia [21,22] and Iran [23]. In Africa, *Candidatus A. camelii* was detected in Hyalomma, Amblyomma, and Rhipicephalus vector ticks in Nigeria and Kenya [25,37] as well in clinical infected camel samples collected during outbreaks of unknown etiology that appeared simultaneously in different Moroccan regions from 2013 onwards. The affected herds showed clinical signs including ventral edematous swelling that spread to the whole body, progressing to complete recumbency and, eventually, death [14].

The situation of anaplasmosis in UAE camels is not fully explored. Investigations using PCR methods did not reveal any DNA from *Cand. M. haemolamae* or *A. marginale* in 55 slightly anemic UAE dromedaries [35]. However, three partial sequences of *Candidatus A. camelii* detected in UAE camels based on analyses using the 16S rRNA gene (GenBank: ON493779.1 and OQ892161.1) or the *groEL* gene (GenBank OQ892162.1) are available in the GenBank nucleotide database (https://www.ncbi.nlm.nih.gov/nuccore, accessed on 5 September 2023) with limited information. In this study, we investigated the presence of *Anaplasma* spp. in dromedary camels in the Abu Dhabi Emirate by PCR targeting *groEL* (which detects both Anaplasma and Ehrlichia) using routine samples received at the Abu Dhabi Agriculture and Food Safety Authority (ADAFSA) veterinary laboratories for Anaplasma testing between 2019 and 2023 to explore Anaplsma infection in camels presenting clinical signs resembling those of a blood parasite infection in other animals. The positive samples were further characterized by partial genome sequencing of the *groEL* gene by ADAFSA and phylogenetic analysis of the identified sequences along with the corresponding sequences of *groEL* gene available in the NCBI nucleotide database. The results of the study provide basic data on the Anaplasma status in the UAE dromedary camel.

## 2. Methods

### 2.1. Sampling

Two hundred eighty-seven whole-blood samples were collected from humped camels (*Camelus dromedarius*) from different regions of the Abu Dhabi Emirate and were received at the veterinary laboratories of Abu Dhabi Agriculture and Food Safety Authority (ADAFSA) for routine diagnosis of Anaplasmosis during the period from 2019 to 2023. We collected 186, 41, and 60 samples from the Abu Dhabi, Al Ain, and Al Dhafra regions, respectively; the total number of samples received per year from the three regions were 20, 19, 40, 105, and 103 for 2019, 2020, 2021, 2022, and 2023, respectively. The data are shown in Table 1. The animals were sampled based on field clinical observation by veterinarians and their tentative suspicion of blood parasite infection due to the detection of similar clinical symptoms as those observed in ruminants with blood parasite infection. The samples were screened for Anaplasmataceae by PCR assay targeting the *groEL* gene, and few positive samples were further characterized by sequencing and phylogenetic analysis of the *groEL* gene sequence.

### 2.2. DNA Extraction

The DNA was extracted from the camels’ whole blood with EDTA using the EZ1 Virus Mini Extraction Kit V2.0 (48) (Qiagen, Hilden, Germany) or the Taco™ Preloaded DNA/RNA Extraction Kit (GeneReach Biotechnology Corporation (GeneReach), Taichung City, Taiwan) according to the manufacturer’s instructions. DNA was stored at −20 °C until amplification.

### 2.3. Polymerase Chain Reaction

All extracted DNA samples were screened by PCR targeting the heat-shock operon ‘*groEL*’ as previously described [21,36]. The Anaplasmataceae-specific PCR primers used were AnaplatF2 5′-GCGTAGTCCGATTCTCCAGT-3′ and AnaGro712R 5′-CCGCGATCAAACTGCATACC-3′ [21,36]. A final 24 μL PCR mix was prepared by adding 1 μL of each primer (10 pmol/μL), 12.5 μL of a master mix from the AmpliTaq Gold^®^ 360 DNA Polymerase Kit (Applied Biosystems, Foster City, CA, USA), 1 μL of 360 GC Enhancer, and 8.5 μL PCR-grade water. Finally, 1 μL of DNA was added to complete the reaction volume to 25 μL and loaded into a Thermocycler Eppendorf Master Cycler^®^ (Eppendorf, Hamburg, Germany). The program was set for an initial denaturation at 95 °C for 8 min, followed by 35 cycles of denaturation at 94 °C for 1 min, hybridization at 59 °C for 40 s, and elongation at 72 °C for 1 min. The final extension step was set at 72 °C for 10 min. A PCR product of 650 bp size was visualized in a 1.8% agarose gel.

### 2.4. Sanger Sequencing

Nine positive PCR products of the heat-shock operon ‘*groEL*’ gene of *Anaplasmataceae* spp. was purified with ExoSAP-IT™ Express PCR Product Cleanup Reagent (Applied Biosystems) and subjected to bidirectional Sanger sequencing at the ADAFSA molecular biology laboratory using the same primers used for the PCR assay. A reaction mixture of 14.5 μL for cycling sequencing was prepared using the BigDye Terminator v3.1 Cycle Sequencing Kit (Applied Biosystems). The reaction mixture consisted of 9 µL of water, 3.5 µL of 5× sequencing buffer, 1 µL of BigDye Terminator V3.1, and 1 µL of 3.2 pmol primer. The purified DNA (5.5 µL) was added. Following the cycling sequencing, the product was purified with the BigDye XTerminator™ Purification Kit (Applied Biosystems) following the manufacturer’s instructions. Sequencing was performed on a SeqStudio Genetic Analyzer (Applied Biosystems) using the ‘LongSeq BDX’ run module. Sequence trimming and assembly were performed with CLC Genomic Workbench v.22 (Qiagen, Aarhus, Denmark), and the obtained consensus sequence was first subjected to GenBank BLAST analysis on the National Center for Biotechnology Information (NCBI) database website (http://www.ncbi.nlm.nih.gov/blast, accessed on 5 September 2023) to identify the organism and compare the sequences obtained to those of reference strains available in the NCBI nucleotide database [31].

### 2.5. Sequence Alignment and Phylogenetic Analysis

For *Anaplasmataceae* spp. characterization by phylogenetic analysis, each sequence of the *groEL* gene was aligned with the corresponding reference sequences of Anaplasma and Ehrlichia species. The analysis also included *groEL* sequences of *Candidatus A. camelii* from the UAE available in GenBank (GenBank accession number ON493779.1). Multiple sequence alignment was performed with the ClustalW program [38] implemented in MEGA software, version 11. A phylogenetic tree was built with the maximum likelihood method and the Kimura 2-parameter model [39] with 1000 Bootstrap confidence using the MEGA software [40].

## 3. Results

### 3.1. Clinical Status of the Sampled Animals

All the sampled animals in this study were tentatively diagnosed to harbor blood parasites infection based on the clinical signs observed by ADAFSA’s veterinary clinicians during field investigations. The main noticed clinical signs in the examined animals were anemia, weakness, fever, anorexia, lymph node hyperplasia with perilymphatic tissue, local, generalized ventral edema, recumbency, and death.

### 3.2. PCR Data

**Figure 1:**
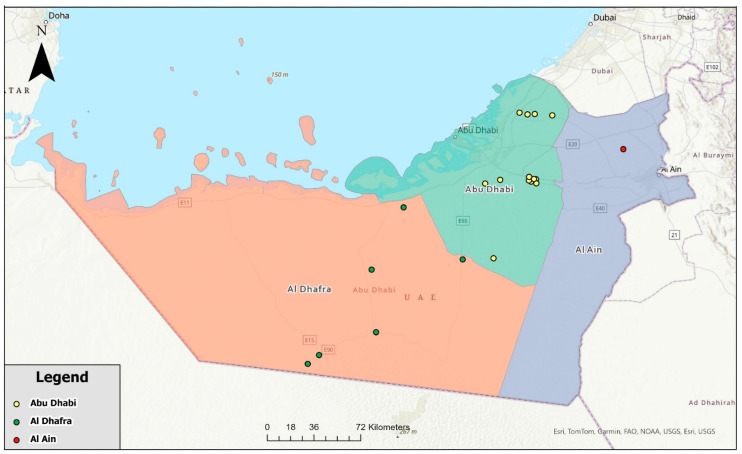
Map of the Abu Dhabi Emirate showing the farms where *Anaplasma* spp. were detected in the three regions (Abu Dhabi farms in yellow color, Al Ain farm in red color, and Al Dhafra farms in green color). 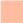 Orange color = Al Dhafra region, 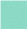 Light green color = Abu Dhabi region and 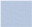 Light purple represents Al Ain region.

**Figure 2:**
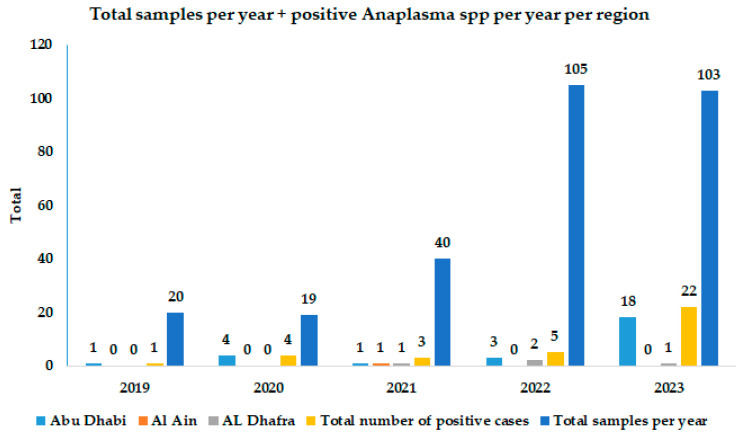
Total number of positive cases of *Anaplasma* spp. across the years 2019–2023 in different regions of the Abu Dhabi Emirate.

Each sample was analyzed by PCR assay targeting the *groEL* gene of Anaplasmataceae. Thirty-five samples 35/287 (12.2%) tested positive for *Anaplasmataceae* spp. The total number of positive cases per year were 1/20 (5%), 4/19 (21.1%), 3/40 (7.5%), 5/105 (4.8%), and 22/103 (21.4%) for 2019, 2020, 2021, 2022, and 2023, respectively. The highest number of reported positive cases was 27/35 (77.1%), from the Abu Dhabi Emirate, followed by 7/35 (20%) form Al Dhafra, and 1/35 (2.9%) from Al Ain regions (Figure 1). The distribution of the positive samples per year, region, and date is shown in Table 1, and the number of positive cases in 2019–2023 in different regions of the Abu Dhabi Emirate is shown in Figure 2.

**Figure 3:**
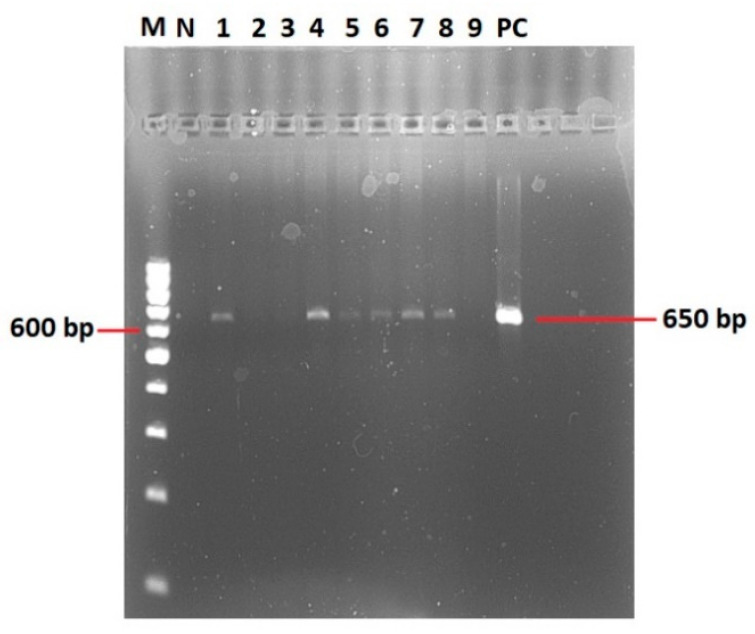
PCR products based on the Anaplasmataceae-specific PCR primers AnaplatF2 and AnaGro712R targeting the *groEL* gene [21,36]. M = DNA marker, N = negative control, PC = positive control; lanes numbered 1–9 contain the samples. Lanes 1–8 contain positive samples for *Anaplasma* spp., while lane 9 contains a negative sample for *Anaplasma* spp.

A representative gel image of the Anaplasmataceae PCR product of 650 bp is shown in Figure 3.

### 3.3. Phylogenetic Analysis

Of the 35 positive samples, only 9/35 (25.7%) samples were sequenced. Those samples were all from the Abu Dhabi Emirate and were collected in 2023. The sequences of the *Candidatus A. camelii groEL* gene obtained in this study were deposited in GenBank under accession numbers from OR159796 to OR159804. These sequences were first subjected to a GenBank BLAST analysis, which confirmed the pathogen identity as *Candidatus A. camelii* (100% identity).

The phylogenetic analysis was performed based on the *groEL* gene sequences of UAE *Candidatus A. camelii* and the corresponding sequences of the reference strains of Anaplasma and Ehrlichia species available in the NCBI GenBank nucleotide database.

**Figure 4:**
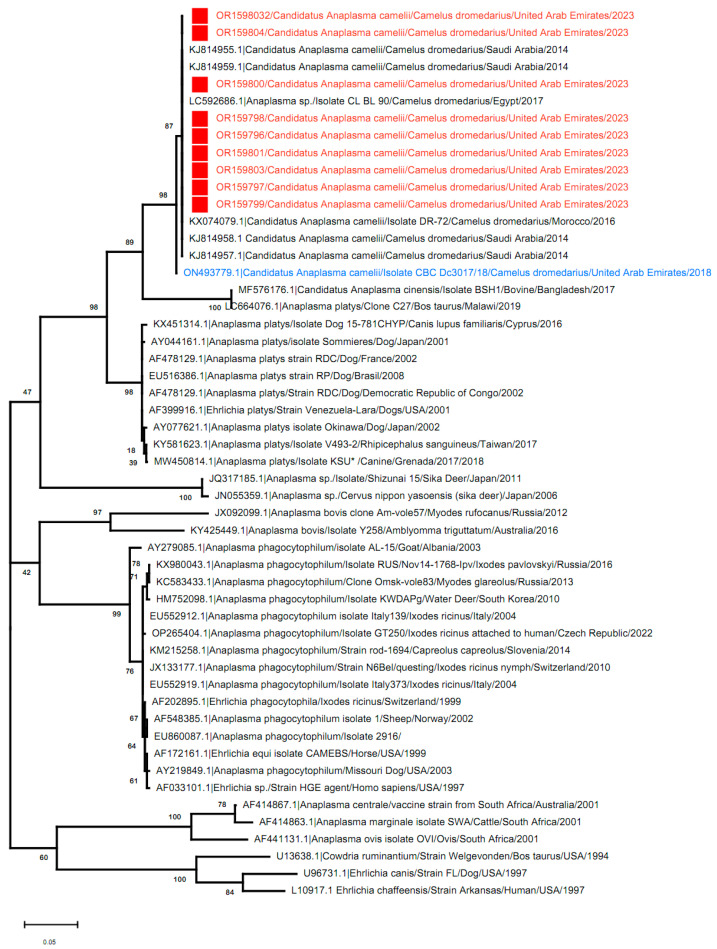
Molecular phylogenetic analysis based on the *groEL* gene of Anaplasmataceae obtained from dromedary camels in the Abu Dhabi Emirate, UAE (showed in red font and labeled with a red square symbol ■) along with corresponding sequences of the *groEL* gene. The *Candidatus A. camelii* sequence from *Camelus dromedarius* previously detected in the UAE (GenBank accession number ON493779.1) is labeled with a blue font. The tree was constructed by the maximum likelihood method. The bootstrap values (1000 replicates) are shown next to the branches. Evolutionary analyses were conducted using MEGA software, version 11.

The results showed that all UAE *Candidatus A. camelii* sequences clustered together with other *Candidatus A. camelii* sequences identified in Saudi Arabia (GenBank accession number KJ814958) and Morocco (GenBank accession number KX074079) as well as with one *Candidatus A. camelii* sequence detected earlier in the UAE (GenBank accession number ON493779.1). This result is supported by a high bootstrap value seen in the phylogenetic tree (Figure 4).

Based on the *groEL* gene sequence analysis, similarity was found to be 100% between all the UAE *Candidatus A. camelii* sequences analyzed in this study and between these sequences and other *Candidatus A. camelii* sequences identified in Saudi Arabia (GenBank accession number KJ814958) and Morocco (GenBank accession number KX074079). However, the new UAE sequences shared 99.5% identity with a *Candidatus A. camelii* sequence previously detected in the UAE (GenBank accession number ON493779.1).

## 4. Discussion

Anaplasmosis in camels is generally described as a subclinical disease [35]. However, symptoms such as weakness, anorexia, emaciation, generalized edema, abortion, lymph node proliferation, anemia, recumbency, and death have been observed in some cases [14,26,27,28]. The sampled animals in this study were tentatively diagnosed to harbor blood parasites infection based on clinical signs including fever, anorexia, local generalized edema, recumbency, and death observed by ADAFSA’s veterinary clinicians.

To the best of our knowledge, this work is the first to describe *Candidatus A. camelii* infection in dromedary camels in the UAE. However, searching of the NCBI GenBank dataset revealed the presence of three partial gene sequences of *Candidatus A. camelii* from the UAE (https://www.ncbi.nlm.nih.gov/nuccore/?term=Candidatus+Anaplasma+camelii++united+arab+emirates, accessed on 5 September 2023). One was a partial sequence of the *groEL* gene (GenBank accession number ON493779.1), while the other two sequences corresponded to the 16S rRNA gene (GenBank accession numbers OQ892162.1 and OQ892161.1) of *Candidatus A. camelii*. This suggests that the pathogen circulates in the region, which denotes the need for additional epidemiological research to ascertain the prevalence and the relevance of the infection, also considering the economic importance of the disease in dromedary camels in the region.

We found 100% identity between the UAE *groEL* gene sequences detected in this study and between these sequences and the *Candidatus A. camelii* sequences detected in Saudi Arabia and Morocco. However, identity was found to be 99.5% with the *Candidatus A. camelii* sequence previously detected in the UAE, suggesting that *Candidatus A. camelii* might comprise independent species and that camels represent the primary reservoir of this pathogen. More in-depth research on the susceptibility of other animal species is needed to understand the host specificity of the pathogen.

The level of infection of Anaplasmataceae varies between different studies conducted in different regions. In the UAE, Anaplasma phagocytophilum was molecularly identified in samples collected from camels showing acute clinical signs ranging from anemia, weakness, fever, anorexia, lymph node hyperplasia with perilymphatic tissue, generalized ventral edema, and recumbency to death [16]. Other researchers in the Taif and Unizah regions of Saudi Arabia recorded more than 30% of Anaplasmataceae infections in camels [21]. In Morocco, 39.62% (42/106) of the camels screened were positive for *Anaplasmataceae* spp., of which 5/42 (2.4%) were characterized as *Candidatus A. camelii* [14]. *Anaplasma* spp. were detected in 111/139 (80%) and 134/139 (96%) samples from Iran according to PCR and nested-PCR sequencing data, and a BLAST search in NCBI GenBank revealed a 100% identity with *Candidatus A. camelii* [23]. However, in this investigation, 12.2% (35/287) of the samples had an Anaplasmataceae signature, with 9 corresponding to *Candidatus A. camelii*. This discrepancy in the infection rates reported by different studies could have been caused by the different sample sizes and may not accurately reflect the global status of Anaplasmataceae, specifically, of *Candidatus A. camelii*. The Anaplasmataceae family includes six genera, i.e., Ehrlichia, Anaplasma, Aegyptianella, Wolbachia, Neorickettsia, and Candidatus Neoehrlichia [5]. However, the Anaplasmataceae PCR assay based on the *groEL* gene targeted only Anaplasma and Ehrlichia [21]. Several studies used the *groEL* gene to discriminate between Anaplasma species variants circulating in camels and *Anaplamsa platys* circulating in dogs, which formed a cluster, and all other forms of Anaplasmosis circulating in different animal species, which formed a separate cluster [14,21,35,41,42]. A limitation of this study is that we only sequenced 9 samples out of the 35 positive samples amplified by the *groEL* gene PCR assay (9/35 = 25.7%) and not the other positive samples (26/35 = 74.3%). The *groEL* gene primers can detect both Anaplasma and Ehrlichia [21]. In a previous study using the same primers, an Ehrlichia strain (closely related to *Ehrlichia canis*) was detected in 3% of the dromedary camels from Saudi Arabia [21]. Therefore, confirmation of infection with *Candidatus A. camelii* is limited only to the nine strains sequenced in this study. Moreover, these samples were from Abu Dhabi and Al Dhafra regions and were collected in 2023; this introduces a further bias to the analysis. Therefore, the presence of Ehrlichia strains and that of other Anaplasma species in camels in other regions of the Abu Dhabi Emirate cannot be excluded without sequencing all amplicons obtained by the *groEL* gene PCR assay.

The vector of *Candidatus A. camelii* was investigated in Morocco and was found to be a Hyalomma sp. [14]. Since this tick has already been characterized in the UAE [43,44], there is a high chance that it may transmit the infection to UAE vulnerable hosts.

A number of Anaplasma species were characterized using 16S rRNA or 23S rRNA PCR assays. For instance, a prevalence of 26% for camel anaplasmosis was found in Saudi Arabia using PCR targeting the 16S rRNA gene and the *groEL* gene. *Anaplasma* spp. identified as *A. platys*-like were present in blood samples taken from camels in Tunisia, with a prevalence of 17.7%, according to PCR (16S rRNA) analysis [36]. Attention should be paid when only a few hypervariable regions of the 16S rRNA gene are used, as this can lead to misclassification of Anaplasma species [45]. Moreover, the 16S rRNA sequences alone remain poorly suited for species discrimination for the genus Anaplasma, as these sequences are very similar, often with identity scores >98% [45]. Therefore, it is recommended to consider other genes, such as *groEL*, which are reliable genetic markers for the taxonomic assignment of Anaplasma species, as gene-specific knowledge is valuable for creating accurate phylogenies [21,45,46]. Anaplasmosis in camels is reported to be a subclinical disease in Tunisian, Indian, and Arabian one-humped camels [35,41,47,48,49,50,51]. Moreover, the camels infected with this pathogen were found to be healthy, without apparent clinical manifestations [21].

In this study, clinical information about the suspected camels sampled for Anaplasma testing was not fully available; however, the sampled camels were suspected to be blood parasite-infected based on a tentative clinical diagnosis by ADAFSA veterinary clinicians. *Candidatus A. camelii* infections detected in camels in different regions of Morocco during simultaneous outbreaks of unknown etiology in 2013 shared the same clinical picture of dependent ventral edema, recumbency, and death [14]. The results of our research, which indicate the spread of the same pathogen in clinically infected camels suspected of being infected with blood parasites, greatly support the hypothesis that this organism has a role in the emergence of such mysterious and fatal clinical cases of unknown etiology and also strongly support the thesis of *Anaplasma* spp. being clinical pathogens in camels. Further research is required on the function of the *Candidatus A. camelii* reservoir or dead-end hosts, which include domestic and wild species of ticks and insects, as well as on the infection mechanism, host target cells, pathogenicity, zoonotic potential, as well as veterinary importance of this microorganism [14]. Studies using in vitro cultures in tick cell lines [52] may help to shed light on this Anaplasma species infection process.

## 5. Conclusions

The molecular results showed the presence of *Candidatus A. camelii* in dromedary camels in the Abu Dhabi region, UAE. To validate the prevalence and veterinary significance of this new pathogen and other *Anaplasma* spp. in the region and to investigate the role of ticks in the transmission of the pathogen, extensive studies involving several camels and ticks in various parts of the UAE are required.

## Figures and Tables

**Table 1:** Distribution of the samples; positive samples received at the laboratory for Anaplasma testing from 2019 to 2023. Positive samples across years and regions are also shown.

Year	Positive Samples/Total per Region			Cumulative Positive Samples per Year	GenBank Accession Number
	Abu Dhabi	Al Ain	Al Dhafra		
2019	1/5 (20%)	0	0/15 (0%)	1/20 (5%)	-
2020	4/18 (22.2%)	0	0/1 (0%)	4/19 (21.1%)	-
2021	1/27 (3.7%)	1/12 (8.3%)	1/1 (100%)	3/40 (7.5%)	-
2022	3/62 (4.8%)	2/29 (6.9%)	0/14 (0%)	5/105 (4.8%)	-
2023	18/74 (24.3%)	0	4/29 (13.8%)	22/103 (21.4%)	OR159796 To OR159804
Total positive samples per region	27/186 (14.5%)	3/41 (4.3%)	5/60 (8.3%)		
Cumulative positive samples				35/287 (12.2%)	
Total samples sequenced	9	-	-	9/35 (25.7%)	

## Data Availability

The partial *groEL* gene sequences generated in this study are available in the NCBI database under the accession numbers mentioned in the manuscript.
